# Isoproterenol Induces Cardiac Injury and Senescence in Sprague–Dawley Rats: A Cost-Effective Pharmacological Model

**DOI:** 10.3390/biomedicines14071445

**Published:** 2026-06-25

**Authors:** Ahmed Altuwaijri, Sarah M. Almufadhili, Taher Hashim Almaki, Dalal Alkhelb, Sultan Almudimeegh, Faris Almutairi, Abdulaziz M. S. Alsaad, Homood M. As Sobeai

**Affiliations:** 1 Department of Pharmacology and Toxicology, College of Pharmacy, King Saud University, Riyadh 11451, Saudi Arabia; 445205614@student.ksu.edu.sa (S.M.A.); dalkhelb@ksu.edu.sa (D.A.); salmudimeegh@ksu.edu.sa (S.A.); faralmutairi@ksu.edu.sa (F.A.); alsaad@ksu.edu.sa (A.M.S.A.); hassobeai@ksu.edu.sa (H.M.A.S.); 2 The Pharmaceutical Care Department, Al Kharj Armed Forces Hospitals, Al-Kharj 16294, Saudi Arabia; 446100125@student.ksu.edu.sa

**Keywords:** cardiac injury, cellular senescence, cardiac remodeling, senolytics, isoproterenol, ageing

## Abstract

**Background/Objectives:** Cardiovascular disease increases with ageing and remains the leading cause of death worldwide. Cellular senescence contributes to cardiac dysfunction in the older population by secreting the senescence-associated secretory phenotype (SASP). Cardiac injury models induced by surgery have been shown to induce senescence in young adult rodents. However, surgical models are complex and associated with high mortality. **Methods:** We established a rat model of injury and senescence using isoproterenol (ISO). Male SD rats received ISO (100 mg/kg) for five days, then hearts were collected on days 10 and 28 after the first ISO dose. **Results:** ISO administration caused cardiac injury, manifested by inflammatory infiltration, fibrosis, and increased cardiomyocyte cross-sectional area. Cardiac injury was accompanied by an increase in the senescence markers SA-β-gal, p16 and p21, and DNA damage marker γH2AX. Moreover, the mRNA levels of p21 increased on day 10, along with several SASP factors, whereas the mRNA levels of p16 increased on day 28. Fibrosis, hypertrophy, and senescence persisted until day 28, indicating long-lasting cardiac remodeling and senescent cell accumulation. **Conclusions:** These findings suggest that ISO can provide a simple, cost-effective platform for studying senescence and cardiac injury. This model facilitates the study of timing, dosage, mechanisms and efficacy of senolytic interventions and may contribute to the development of senescence-targeted therapies.

## 1. Introduction

Population aging is a major global health challenge, accompanied by a rapidly increasing burden of cardiovascular diseases (CVDs) [1,2]. Recent cardiac interventions including revascularization and pharmacological treatment have dramatically improved cardiac health [3,4]. However, heart failure remains a progressive condition and no therapy can fully restore cardiac function. Therefore, there is a need to identify and target new biological mechanisms that drive cardiac deterioration.

Cellular senescence, a hallmark of aging, contributes to cardiovascular deterioration by secreting proinflammatory factors collectively known as the senescence-associated secretory phenotype (SASP) [5,6]. These factors promote chronic inflammation, fibrosis, and secondary senescence in surrounding cells, thereby exacerbating tissue dysfunction and impairing cardiac repair [6,7,8]. Cardiac senescence was also found in young adult rodents exposed to ischemia–reperfusion injury (IRI), doxorubicin, and angiotensin II [9,10,11,12,13,14,15]. Importantly, senolytics including navitoclax and the dasatinib and quercetin combination (D+Q) improved cardiac function in young adult and aged cardiac-injured mice [9,10,12,16]. These findings warrant further investigation into how senescence affects cardiac injury, how senescence clearance improves cardiac outcomes, when to administer senolytics, and which dose and drug are most effective.

Isoproterenol (ISO) is a non-selective β-adrenergic agonist that stimulates both β1- and β2-adrenergic receptors [17]. It is commonly used to induce cardiac injury in murine models by overstimulating the heart [18]. High ISO dose sharply increases the inotropic and chronotropic activity of the heart. As a result, oxygen demand increases significantly, leading to hypoxia. Moreover, ISO has a short half-life (2–5 min), allowing controlled and reproducible induction of injury and minimizing off-target effects [19]. However, ISO is dose-dependent. For example, a single acute dose has been used as a model of Takotsubo cardiomyopathy and type 2 MI [20,21]. In contrast, high repeated ISO dosing does not correspond to a specific cardiovascular disease in humans but results in persistent cardiac remodeling that resembles aspects of heart failure. However, some studies suggest that high doses of ISO can reproduce similar consequences to myocardial infarction in humans, including hypoxia, necrosis, fibrosis, hypertrophy, and disruption of cardiac function [17,18,22,23,24].

Left anterior descending (LAD) coronary artery ligation, Transverse Aortic Constriction (TAC), and IRI are the common surgical cardiac models that more closely mimic myocardial infarction or pressure overload-induced heart failure [25]. However, these models require skilled researchers, cause suffering and distress to the animals, and have high mortality rates. Furthermore, opening the chest of the animal adds an extra variable to the research that does not occur in human cases of MI or other cardiac injuries. Therefore, we aimed to provide an additional model to study senescence and cardiac remodeling.

In this study, we hypothesized that ISO would induce senescence, providing a simple, cost-effective cardiac injury model that facilitates the study of senescence and cardiovascular disorders. We further aimed to show that the ISO injury is persistent for 28 days, allowing long-term evaluation of senolytics and senomorphics.

## 2. Materials and Methods

### 2.1. Animals and Procedures

Male Sprague–Dawley rats were used in this experiment and were obtained from the Animal Care Center at the College of Pharmacy, King Saud University (Riyadh, Saudi Arabia). Rats were housed under conditions of 25 °C, 50% relative humidity, and a 12 h light/dark cycle. They had free access to standard laboratory chow and water.

All animal procedures were conducted following the National Institutes of Health guidelines for the Care and Use of Laboratory Animals. Before conducting the experiments, ethical approval (KSU-SE-25-72) was obtained from the Research Ethics Committee (REC) at King Saud University.

#### 2.1.1. Isoproterenol Administration

10–12-week-old male Sprague-Dawley rats were randomly selected and subcutaneously administered 100 mg/kg isoproterenol (ISO; I5627, Sigma-Aldrich, St. Louis, MO, USA) (*n* = 20) or saline (*n* = 20) for five consecutive days. This selected dose has been shown to induce cardiac injury and reduce cardiac function [26]. ISO was dissolved in saline at a final concentration of 50 mg/mL. According to the animal’s weight, 400–800 µL were injected subcutaneously. Next, rats were culled either on day 10 or day 28 after the first ISO dose. The final groups were: Saline Day 10 (*n* = 10), Saline Day 28 (*n* = 10), ISO Day 10 (*n* = 10), and ISO Day 28 (*n* = 10).

Day 10 was selected because previous studies and our observations showed that senescence peaks around day 10 after cardiac injury [10]. In addition, senolytics have previously been administered approximately 72 h after cardiac injury in young adult rodents because senescence plays an important role in limiting fibrosis during the early phase of cardiac repair. [10,27]. Day 28 was determined to assess long-term remodeling and to ensure that rats do not spontaneously recover. Moreover, several studies reported beneficial effects of senolytics on cardiac remodeling by day 28 after injury [10,16] (detailed in the Discussion).

#### 2.1.2. Heart Collection

The rats were humanely euthanized by Schedule 1 cervical dislocation, and the chest was opened. Next, 30 mM potassium chloride was injected into the LV to arrest the heart in diastole. After that, the right atrium was cut, and the heart was slowly perfused by injecting phosphate-buffered saline (PBS) into the LV to remove the blood. Then, the heart was excised from the chest cavity and sectioned into base, mid, and apex. These were either directly frozen in liquid nitrogen or fixed in 10% formalin or placed in Tissue-Tek OCT Compound (Sakura Finetek, Tokyo, Japan) and snap frozen on a cold metal surface that was placed in liquid nitrogen. Rats’ body weight was measured before culling, and the heart weight was measured before cutting into the base, mid, and apex.

### 2.2. Sample Processing and Tissue Cutting

Samples intended for paraffin-embedded sections were fixed in 10% neutral buffered formalin for 24–48 h. After that, samples were immersed in 70% ethanol and kept at 4 °C until processing. The samples were processed using Tissue-Tek VIP 5 Jr E2 tissue processor (Sakura Finetek, Tokyo, Japan). Processing included dehydration using an increasing concentration of industrial methylated spirit (90%, then 100%) and clearing in xylene. Next, samples were embedded in paraffin wax (Sakura Finetek, Tokyo, Japan), cut into 3 µm (RM2245, Leica Biosystems, Nussloch, Germany) thick sections, mounted on super frost slides (LVQ075W, Labvida Scientific, Nanjing, China), and stored at 4 °C. On the other hand, frozen sections were cut at 8 µm using a Cryostat (CM1850, Leica Biosystems, Nussloch, Germany), mounted on super frost slides (LVQ075W, Labvida Scientific, Nanjing, China), and stored at −80 °C.

### 2.3. Immunohistochemistry and Immunofluorescence

#### 2.3.1. Dewaxing and Antigen Retrieval for Paraffin-Embedded Sections

For staining of paraffin-embedded sections, the sections were dewaxed in an oven at 65 °C for 30 min, then placed in xylene for 5 min, followed by rehydration through graded ethanol solutions (100%, 90%, and 70%; 5 min each). To perform antigen retrieval, slides were placed in 1 mM citric acid (pH 6.2) for 15 min in a microwave.

#### 2.3.2. Staining for H&E and Masson’s Trichrome

Slides were placed for 3 min in Mayer’s hemalum solution (109249, Sigma-Aldrich, St. Louis, MO, USA) then washed using tap water. After that, slides were immersed in eosin (109844, Sigma-Aldrich, St. Louis, MO, USA) for 1–2 min, then washed in tap water before dehydration and mounting.

Masson’s trichrome kit (Ab150686, Abcam, Cambridge, UK) was used, and the manufacturer’s protocol was followed. Slides were placed in Bouin’s solution for one hour at 60 °C, and then washed in tap water. Next, the slides were stained with haematoxylin for 5 min, and then washed using tap water. After that, Biebrich Scarlet/Acid Fuchsin Solution was applied to the tissues for 10 min. Next, phosphomolybdic/phosphotungstic acid solution was applied for 15 min. Next, the Aniline Blue solution was applied for 10 min. Finally, the slides were washed with distilled water, then immersed in acetic acid Solution (1%) for 3–5 min. For H&E and MT, the slides were dehydrated in the oven for 30 min and then cleared in xylene and mounted with DPX.

#### 2.3.3. Staining for p21, γH2AX, and Wheat Germ Agglutinin (WGA)

Sections were dewaxed and rehydrated, followed by antigen retrieval. After that, sections were incubated overnight at 4 °C with antibodies against p21 (1:300, ES3100, ELK Biotechnology, Wuhan, China), or γH2AX (1:500, AP0687, ABclonal, Wuhan, China). After incubation, sections were washed with TBS and incubated with ABflo^®^ 488 secondary (1:300, AS053, ABclonal, Wuhan, China) and DAPI (0.5–1 µg/mL, C0060, Solarbio, Beijing, China) for two hours. Sections were then washed with TBS and mounted.

For the WGA staining, sections were incubated with WGA (1:100, W11262, Thermo Fisher Scientific, Waltham, MA, USA) and DAPI (C0060, Solarbio, Beijing, China) for two hours. Then, sections were washed with TBS and mounted. All antibodies were diluted in PBS containing 2% BSA and 0.05% Triton X-100 and mounted using an antifade fluorescence mounting medium (S2100, Solarbio, Beijing, China).

#### 2.3.4. SA-β-gal and p16 Staining

Frozen sections were used for SA-β-gal (HY-K1089, MedChemExpress, Monmouth Junction, NJ, USA) and p16 staining. Frozen sections were removed from −80 °C and kept at room temperature to dry for five minutes.

SA-gal staining: sections were fixed using the fixation solution in the kit for 10 min. Next, sections were washed with TBS and incubated with the β-galactosidase staining solution (pH = 6) at 37 °C for 24–30 h. Finally, sections were washed with TBS and mounted using an antifade fluorescence mounting medium (S2100, Solarbio, Beijing, China).

p16 staining: sections were fixed using 10% neutral buffered formalin, washed using TBS, and incubated overnight at 4 °C with antibody against p16 (1:500, K010139P, Solarbio, Beijing, China). On the next day, sections were washed using TBS and incubated with ABflo^®^ 488 secondary (1:300, AS053, ABclonal, Wuhan, China) and DAPI (0.5–1 µg/mL, C0060, Solarbio, Beijing, China) for two hours at room temperature. Sections were then washed with TBS and mounted using antifade fluorescence mounting medium (S2100, Solarbio, Beijing, China).

### 2.4. Imaging and Analysis

Images of H&E, SA-β-gal, and Masson’s trichrome were captured using a Nikon Eclipse 80i light microscope equipped with a DXM1200C digital camera (Nikon Corporation, Tokyo, Japan). Images of p16, p21, γH2AX, and WGA were captured using a Zeiss Axio Observer Z1 epifluorescence microscope (Carl Zeiss, Oberkochen, Germany) with Zen Pro software (version 2.5). A minimum of ten images over two sections were captured at 40× magnification for each stain. The percentage of H&E, SA-β-gal, and Masson’s trichrome was measured by dividing the positive area by the whole tissue in the same image. p16, p21, γH2AX, positive cells were manually counted and divided by the number of DAPI-positive cells on the same image. Cardiomyocyte cross-sectional area was measured using ImageJ software (version 1.54p) following these steps: first, contrast enhancement; second, Gaussian smoothing (σ = 2); third, Otsu thresholding; fourth, binary conversion and hole filling; and fifth, watershed segmentation. Particles between 200 and 1500 µm^2^ were analyzed. A minimum of 2000 cells was measured for each rat. The ImageJ software (version 1.54p) was used to analyze and quantify the images in all experiments. qPCR along with histological assessments and imaging of H&E and Masson’s trichrome were conducted in a blinded manner; immunofluorescence analyses were performed without blinding.

### 2.5. Gene Expression Analysis (RT-qPCR)

Total RNA was extracted by homogenizing the tissue in TRIzol (15596026, Invitrogen, Carlsbad, CA, USA). Next, the RNA was purified using the HavenSci RNA kit (RE95050, HavenSci, Thuwal, Saudi Arabia). The purity of the RNA was determined using NanoDrop™ 8000 Spectrophotometer (Thermo Fisher Scientific, Waltham, MA, USA). cDNA at 1 µg/µL was synthesized following the manufacturer’s instructions using the PCR3005 kit (HavenSci, Thuwal, Saudi Arabia).

EverGreen Universal Real-Time PCR Master Mix (PCR5505, HavenSci, Thuwal, Saudi Arabia) was used to quantify gene expression on the Applied Biosystems 7500 Fast Real-Time PCR System. The ΔΔCt method [28] was used to calculate relative gene expression. Primers were internally designed and synthesized by HavenSci (Table 1).

### 2.6. Statistical Analysis

The Shapiro–Wilk normality test was used to determine whether the data were normally distributed and whether to use parametric or non-parametric tests. Differences between groups on the two time points were analyzed using a two-way ANOVA followed by Šídák’s multiple comparisons. Data are presented as mean ± SD and statistical significance was set at α = 0.05. Statistical analyses were conducted using GraphPad Prism (version 9).

## 3. Results

### 3.1. ISO Induces Cardiac Injury and Fibrosis in the Apex of Young Adult Rats

**Figure 1:**
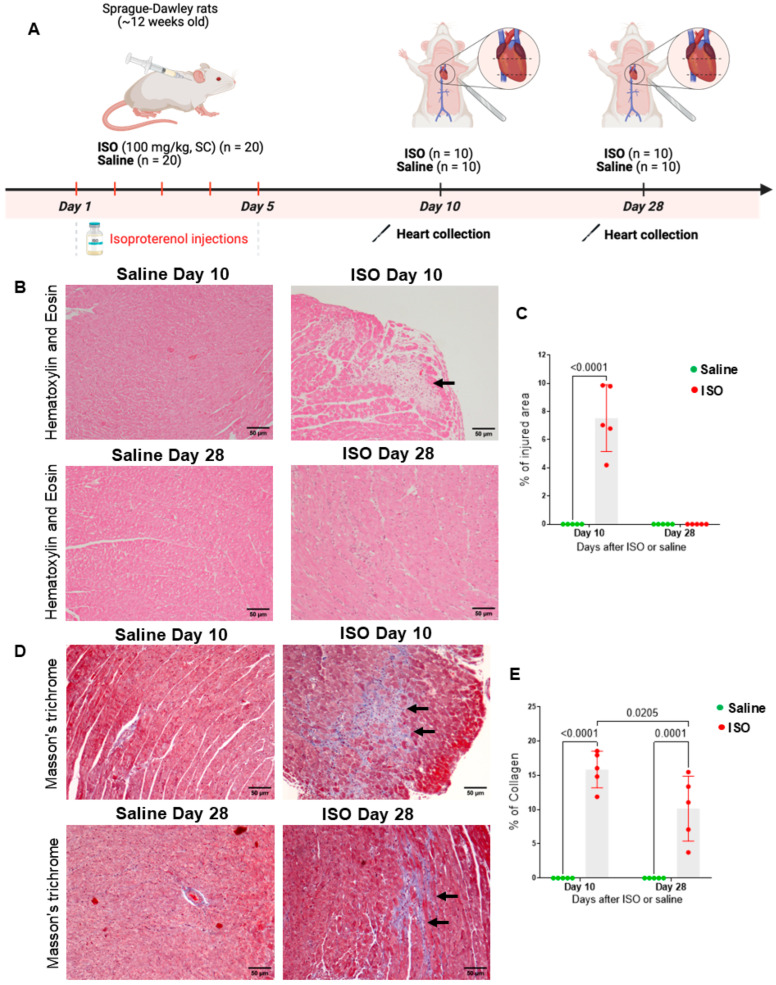
ISO induces inflammatory cellular infiltration and collagen deposition in the apex of the young adult rat. (**A**) 10–12-week-old rats were administered 100 mg/kg ISO or saline for five days, and then apexes were collected on days 10 and 28. (**B**) H&E-stained cardiac sections. Black arrows indicate areas of inflammatory cell infiltration. (**C**) Quantification of the injured area. (**D**) MT-stained cardiac sections. Black arrows indicate areas of collagen deposition. (**E**) Quantification of collagen-positive area. Scale bars = 50 µm. Data are expressed as the mean ± S.D. Statistical analysis was performed using two-way ANOVA followed by Šídák’s multiple comparisons test. *n* = 5 per group. Panel (**A**) was created using BioRender (As Sobeai, H. (2026). https://biorender.com/q0u7o5z, accessed on 21 May 2026).

Experimental design, timeline, and animal groups were shown in Figure 1A. To assess whether ISO (100 mg/kg) for five consecutive days successfully induces cardiac injury, we stained for H&E and MT to measure cellular infiltration and collagen deposition.

H&E staining showed a significantly increased (*p* < 0.05) cellular infiltration in the ISO-injured rats compared to saline on day 10 after the first ISO dose (post-ISO) (Figure 1C). In contrast, cellular infiltration was not detected on day 28 post-ISO. Masson’s trichrome staining revealed significantly increased (*p* < 0.05) collagen deposition in the ISO-injured rats on day 10 and day 28 post-ISO compared to saline (Figure 1E).

### 3.2. Effect of ISO on Cardiac Hypertrophy

**Figure 2:**
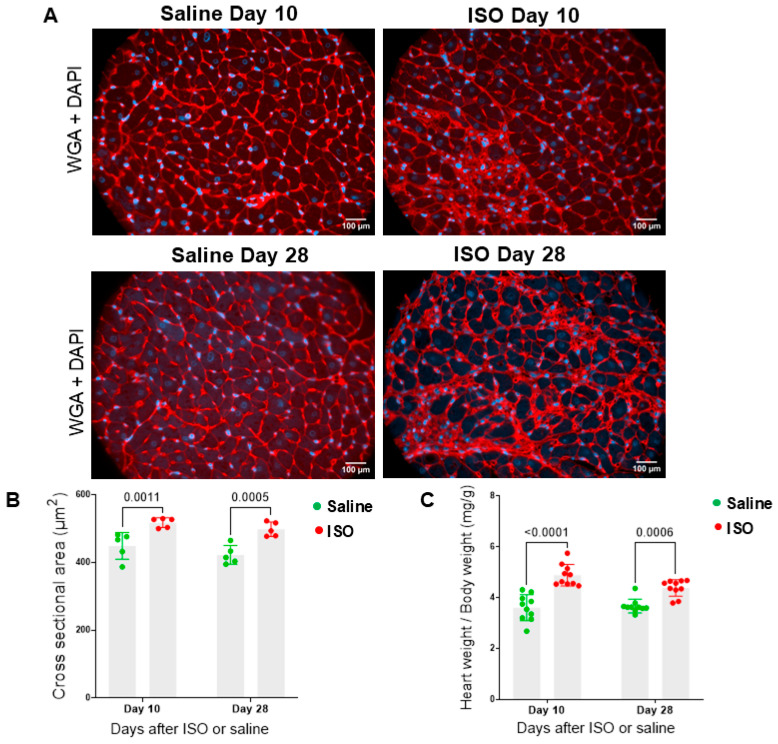
ISO increases hypertrophy in the apex of the young adult rat. 10–12-week-old rats were administered 100 mg/kg ISO or saline for five days, and then apexes were collected on days 10 and 28. (**A**) Representative images of WGA and DAPI staining. (**B**) Quantification of cardiomyocyte cross-sectional area. (**C**) Quantification of heart weight/body weight ratio. Scale bars = 100 µm. Data are expressed as the mean ± S.D. Statistical analysis was performed using two-way ANOVA followed by Šídák’s multiple comparisons test. *n* = 5 per group for cross-sectional area measurements, and *n* = 10 per group for HW/BW measurements.

We then measured cardiac hypertrophy using cardiomyocyte cross-sectional area and heart weight–to–body weight ratio. Both parameters were significantly increased (*p* < 0.05) in the ISO-injured rats on day 10 and day 28 post-ISO compared to saline (Figure 2).

### 3.3. Effect of ISO on Cardiac Senescence and SASP Factors

**Figure 3:**
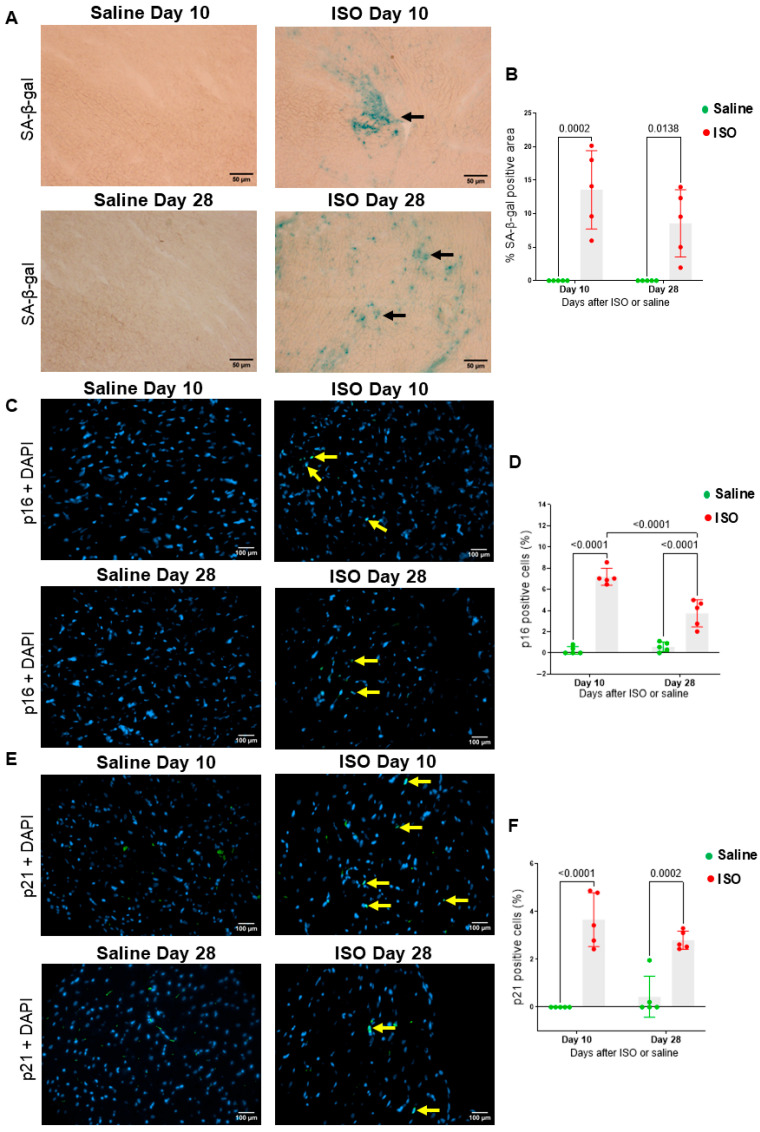
ISO induces senescence in the apex of the young adult rat. 10–12-week-old rats were administered 100 mg/kg ISO or saline for five days, and then apexes were collected on days 10 and 28. (**A**) Representative SA-β-gal staining images (blue; black arrows). (**B**) Quantification of SA-β-gal-positive area. (**C**) Representative p16 staining images (green; yellow arrows). (**D**) Quantification of p16-positive cells. (**E**) Representative p21 staining images (green; yellow arrows). (**F**) Quantification of p21-positive cells. Scale bars = 100 µm. Data are expressed as the mean ± S.D. Statistical analysis was performed using two-way ANOVA followed by Šídák’s multiple comparisons test. *n* = 5 per group.

To investigate whether cardiac injury induced by ISO is accompanied by senescence, we stained cardiac tissues for SA-β-gal, p16, and p21. ISO treatment significantly increased (*p* < 0.05) p16, p21, and SA-β-gal on day 10 and day 28 post-ISO compared to saline (Figure 3A–F).

**Figure 4:**
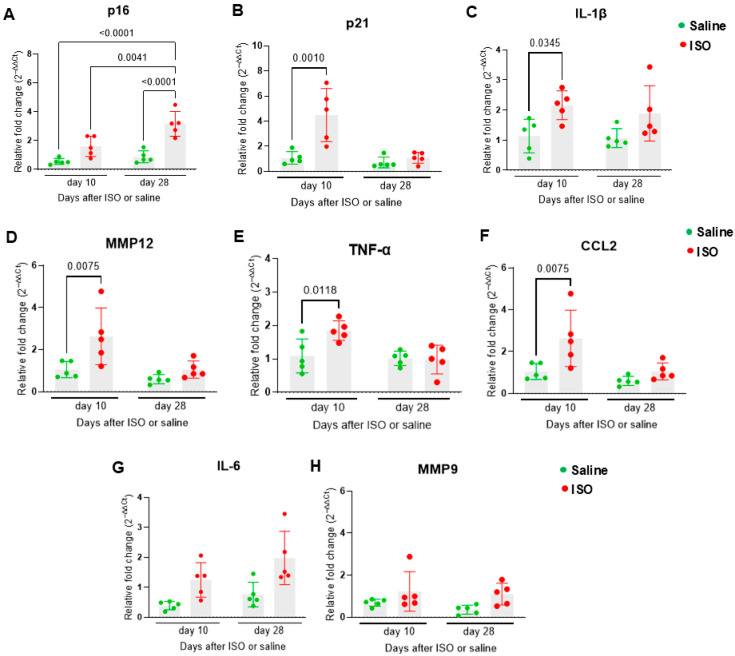
Changes in senescence markers and SASP factors in the apex of the young adult rat after ISO or saline. 10–12-week-old rats were administered 100 mg/kg ISO or saline for five days and then apexes were collected on days 10 and 28. RT-qPCR analysis of (**A**) p16, (**B**) p21, (**C**) IL-1β, (**D**) MMP12, (**E**) TNF-α, (**F**) CCL2, (**G**) IL-6, and (**H**) MMP9. The mRNA levels were normalized to Actb mRNA levels within the same samples and then expressed as the fold change in the experimental group relative to the equivalent control group using the ΔΔCt method. Data are expressed as the mean ± S.D. Statistical analysis was performed using two-way ANOVA followed by Šídák’s multiple comparisons test. *n* = 5 per group.

To further assess senescence, we measured the mRNA levels of senescence markers (p16 and p21) and common SASP factors (IL-1β, MMP12, TNF-α, CCL2, IL-6, and MMP9). The mRNA levels of p16 increased (*p* < 0.05) in the ISO-injured young adult rats on day 28 compared to day 10 and to saline groups (Figure 4A). In contrast, the mRNA levels of p21 were increased (*p* < 0.05) on day 10 in the ISO-injured rats compared to saline but returned to baseline on day 28 (Figure 4B). Similarly, the mRNA levels of IL-1β, MMP12, TNF-α, and CCL2 increased (*p* < 0.05) in the ISO-injured young adult rats on day 10 compared to saline, and then returned to baseline on day 28 (Figure 4 C–F). However, ISO injury did not affect the mRNA levels of IL-6 or MMP9 on either day 10 or day 28 (Figure 4G,H).

### 3.4. Effect of ISO on DNA Damage

**Figure 5:**
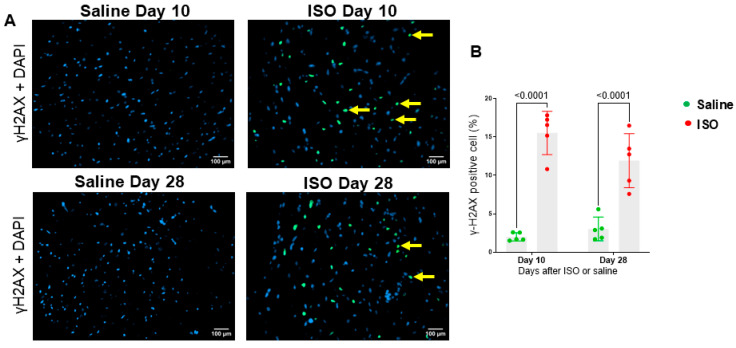
ISO induces DNA damage in the apex of the young adult rat. 10–12-week-old rats were administered 100 mg/kg ISO or saline for five days, and then apexes were collected on days 10 and 28. (**A**) Representative γH2AX staining images (green; yellow arrows). (**B**) Quantification of γH2AX-positive cells. Scale bars = 100 µm. Data are expressed as the mean ± S.D. Statistical analysis was performed using two-way ANOVA followed by Šídák’s multiple comparisons test. *n* = 5 per group.

To investigate the mechanisms involved in senescence induction, we assessed DNA damage by measuring γH2AX expression. γH2AX levels increased (*p* < 0.05) in ISO-injured rats on days 10 and 28 compared to saline (Figure 5).

## 4. Discussion

Isoproterenol administration in this study resulted in cardiac injury accompanied by cellular senescence. This established a simple cost-effective pharmacological platform for investigating cardiac senescence and remodeling. ISO administration (100 mg/kg for five consecutive days) induced inflammatory infiltration, collagen deposition, and cardiomyocyte hypertrophy without mortality, enabling long-term evaluation of injury progression and senescence dynamics.

ISO is commonly used to induce cardiac injury. Consistent with the literature, ISO in this study induced cellular inflammation, collagen deposition, and cardiomyocyte hypertrophy [17,24,29,30]. However, in this study, we specifically focused on the left ventricular apex, as this region is more susceptible to ISO-induced cardiac injury due to the higher density of β-adrenergic receptors compared with the mid and basal regions [31]. Consistently, a study showed that ISO induces functional impairment predominantly in the apical region, with minimal changes in the base [32]. At the histological level, mononuclearcell infiltration and collagen deposition were observed in the apex but not in the base [32]. Therefore, focusing on the apex is an advantage of this study and may partially explain the variability reported in the outcomes of ISO experiments across the literature [33]. Moreover, in this study, rats were followed for 28 days, whereas most previous studies evaluated outcomes within 14 days after the first ISO administration [29,30,34,35,36,37,38].

ISO in this study induced senescence, manifested by increased protein expression of p16 and p21 on days 10 and 28 after the first ISO dose. However, the mRNA levels of p16 increased on day 28 but not on day 10. In contrast, p21 increased on day 10 but returned to baseline levels on day 28 in the ISO-injured rats. This discrepancy may have resulted from post-transcriptional and post-translational regulation of p21 and p16, including modulation of mRNA stability, translation efficiency, and proteasome-dependent degradation, which may lead to sustained protein accumulation despite transcription declines [39].

In line with our findings, various forms of cardiac injury have been reported to induce senescence characterized by increased p16, p21, and SA-β-gal. For example, p16, p21, and SA-β-gal increased in young adult mice exposed to ischemia–reperfusion injury (IRI), doxorubicin, and angiotensin II [9,10,11,12,13,14,15]. Compared with surgical cardiac injury models, the ISO model provides a pharmacological approach that induces senescence while avoiding surgical procedures and their associated complications. ISO mechanism shares key pathological features with rodent models that try to mimic myocardial infarction, including IRI and LAD ligation. These models induce myocardial damage primarily through ischemia and hypoxia. However, ISO injury occurs in the absence of coronary artery occlusion. Compared to C57BL/6 mice, we observed that collagen is present on day 28 after ISO-injured rats while C57BL/6 mice showed fibrosis resolution on 28 days after the last ISO dose [40]. Together, these findings highlight the advantages of using the ISO rat model to investigate cardiac remodeling and senescence.

In this study, we showed that DNA damage increased in the ISO-injured rats. ISO causes hypoxia, oxidative stress and mitochondrial dysfunction leading to DNA damage which induces senescence regardless of telomere shortening [7,41,42,43]. Consistently, in vitro exposure of cardiomyocytes and cardiac fibroblasts to hypoxia or oxidative stress has also been shown to induce senescence markers, including increased p21, p16, and SA-β-gal activity [15].

Measuring DNA damage was preferred over measuring oxidative stress because oxidative stress markers fluctuate within hours to several days and two time points will not capture the effect of oxidative stress [44,45]. For example, one study administered 85 mg/kg ISO (s.c.) to young adult rats for two days and then sacrificed them after the last dose. They found that both SOD and catalase levels were elevated in the ISO-treated rats [46]. In contrast, another study administered 100 mg/kg ISO (s.c.) for two days and sacrificed the animals 12 h after the last dose. They found that SOD and catalase levels were decreased [45]. In addition, another study administered a small ISO dose (3 mg/kg) (s.c.) to mice for three days. They found increased SOD levels and decreased catalase levels [47]. Therefore, DNA damage represents a more stable indicator of cellular stress and senescence compared with oxidative stress measurements.

The mRNA expression of IL-1β, MMP12, TNF-α, and CCL2 increased on day 10 in ISO-injured rats compared to saline. However, they returned to normal on day 28. On the other hand, no difference was found in mRNA expression of IL-6 and MMP9 between groups on days 10 and 28.

IL-1β and TNF-α are inflammatory mediators linked with the acute injury phase. In line with our findings, ISO increased the protein levels of IL-1β and TNF-α in young adult rats [48,49]. In addition, both were shown to increase with senescence after cardiac injury [7,50]. Moreover, IL-1β and TNF-α were reported to drive senescence by modulating pathways such as NF-κB activation, p53-p21 induction, and oxidative stress in cardiac cells [51,52,53]. CCL2 is a chemokine that promotes monocyte/macrophage recruitment to the injury site, while MMP12 is involved in the extracellular matrix remodeling. Consistently, studies showed that cardiac injury induced by ISO or IRI increased MMP12 and CCL2 [10,54]. Both are involved in early cardiac injury remodeling and have been identified as SASP factors [7,10,55]. Therefore, IL-1β, TNF-α, CCL2, and MMP12 are likely to contribute to senescence development and cardiac remodeling. However, it is hard to determine whether these factors are produced from senescent cells or if they are a result of an early remodeling response.

Although IL-1β, MMP12, TNF-α, and CCL2 transcripts were elevated on day 10, they returned to baseline by day 28, whereas senescence markers remained increased. This divergence is consistent with senescence being a stable cell state while the SASP is dynamic and can be highest during the early post-injury inflammatory phase, then attenuate as inflammation resolves and remodeling becomes the dominant pathology [56]. Moreover, measuring mRNA levels does not necessarily reflect the protein levels of the SASP and additional timepoints are vital to understand SASP patterns [57]. Measuring SASP on different time points and at the protein/secretome level (e.g., multiplex cytokine assays) and/or expanding the SASP panel would further strengthen the characterization of SASP dynamics in this model.

Contrary to the literature, ISO did not affect the mRNA expression of IL-6 and MMP9 [58,59,60,61,62,63]. The absence of changes in IL-6 and MMP9 may indicate selective SASP activation in this model. However, it may also be caused by differences in time of sample collection, tissue region analyzed, or species/model-specific responses reported across the literature.

Day 28 was selected as an endpoint because various studies have reported positive outcomes on day 28 after injury when using senolytics [9,10,12]. Furthermore, we aimed to show that rats are not able to recover spontaneously, which facilitates investigating interventions to improve cardiac outcomes.

Day 10 was chosen because senolytics are usually administered 2–4 days after injury in young cardiac injury models [10]. A study showed that senescence has a beneficial early role in limiting collagen deposition after cardiac injury [27]. Therefore, early senolytic administration may interfere with the limitation of collagen deposition. However, senescent cells may persist in the heart after injury, creating an unfavorable environment by producing SASP factors [64]. Moreover, based on our observations, senescence peaked on day 10 compared with day 7 and day 14 in ISO-injured young adult mice. Similarly, in the IRI model, SA-β-gal levels peaked at 72 h after injury compared to 24 h and 1 week [10].

Our findings demonstrate that ISO induces cardiac injury and senescence. However, several limitations should be considered for future studies. First, functional assessments were not conducted in this study due to the lack of facilities. Therefore, while we showed cardiac injury and remodeling, we cannot evaluate the impact of these structural changes on cardiac function. Second, expanding senescence assessment by including additional markers such as Lamin B1 loss or telomere-associated DNA damage foci (TAFs), together with a broader panel of SASP factors, would further strengthen the characterization of senescence. Moreover, measuring the protein levels of SASP factors at additional time points using ELISA or multiplex cytokine assays would help elucidate their patterns. Third, the specific cardiac cell populations undergoing senescence were not identified in this study. Senescence markers were assessed at the tissue level and therefore may originate from multiple cardiac cell types, including cardiomyocytes, fibroblasts, endothelial cells, and infiltrating immune cells [7,65,66,67]. Future studies using co-localization of senescence markers with cell-type-specific markers will be important to determine the relative contribution of individual cardiac cell populations to senescence and remodeling in this model.

An additional limitation of this study is that only male rats were included. Male animals were selected to minimize variability associated with the estrous cycle and to establish a reproducible model of ISO-induced cardiac injury and senescence. In addition, as this represents a relatively new exploratory model, we aimed to reduce biological variability as much as possible during its initial characterization. Furthermore, some studies have suggested that male rodents are more susceptible to ISO injury. It has been reported that male rodents exhibit greater mortality, fibrosis, hypertrophy, and β-adrenergic receptor activation compared with females [68,69,70,71]. However, future studies should include both male and female animals to determine whether the cardiac senescence and injury responses observed in the present study are influenced by sex and to improve the translational relevance of this model.

## 5. Conclusions

Collectively, our findings demonstrate that ISO administration induces cardiac injury and cellular senescence in young adult male SD rats. This model may offer a simple, cost-effective preclinical model for studying the temporal dynamics of cardiac senescence. It may also serve as a platform for investigating senescence-targeted interventions and for exploring the relationship between cardiac disease and senescence.

## Figures and Tables

**Table 1:** List of forward and reverse primers used in RT-qPCR.

Gene	Forward Primer	Reverse Primer	NM Number
*p16 (Cdkn2a)*	GTCACCGACAGGCATAACTT	TGAGCAGAAGTTATGCCTGTC	NM_031550.1
*p21 (Cdkn1a)*	TCAGACCTGTGAAGATCCTTTG	GAGCAGCAGATCACCAGATT	NM_080782.4
*IL-6*	CTTCACAAGTCGGAGGCTTAAT	GCATCATCGCTGTTCATACAATC	NM_012589.2
*IL-1* *β*	TGACCCATGTGAGCTGAAAG	CGTTGCTTGTCTCTCCTTGTA	NM_031512.2
*TNF-α*	GCAGATGGGCTGTACCTTATC	GAAATGGCAAATCGGCTGAC	NM_012675.3
*Mmp9*	GATCCGCAGTCCAAGAAGAT	CTGAGCCTAGACCCAACTTATC	NM_031055.2
*Mmp12*	CTGGTTCGGTTGTTAGGAAGA	CCCTGAGCATACAGTGGATATG	NM_053963.2
*CCL2 (MCP-1)*	GGAATGGGTCCAGAAGTACATTAG	GCTGAAGTCCTTAGGGTTGATG	NM_031530.1
*β-Actin (Actb)*	TGTGACGTTGACATCCGTAAAG	GGCAGTAATCTCCTTCTGCATC	NM_031144.3

Abbreviations: *Cdkn2a*, cyclin-dependent kinase inhibitor 2A; *Cdkn1a*, cyclin-dependent kinase inhibitor 1A; *IL-6*, interleukin-6; *IL-1β*, interleukin-1 beta; *TNF-α*, tumor necrosis factor alpha; *Mmp9*, matrix metalloproteinase-9; *Mmp12*, matrix metalloproteinase-12; *CCL2*, C–C motif chemokine ligand 2; *Actb*, beta-actin.

## Data Availability

The data supporting the findings of this study are available from the corresponding author upon reasonable request.

## Abbreviations

The following abbreviations are used in this manuscript:

Actb	Beta-actin
ANOVA	Analysis of variance
BSA	Bovine serum albumin
CCL2	C-C motif chemokine ligand 2
CM-CSA	Cardiomyocyte cross-sectional area
CVDs	Cardiovascular diseases
DAPI	4′,6-diamidino-2-phenylindole
DPX	Dibutylphthalate polystyrene xylene
H&E	Hematoxylin and eosin
HW/BW	Heart weight-to-body weight ratio
IL-1β	Interleukin-1β
IL-6	Interleukin-6
IRI	Ischemia–reperfusion injury
ISO	Isoproterenol
Mmp9	Matrix metalloproteinase 9
Mmp12	Matrix metalloproteinase 12
p16	Cyclin-dependent kinase inhibitor 2A (CDKN2A)
p21	Cyclin-dependent kinase inhibitor 1A (CDKN1A)
TNF-α	Tumor necrosis factor α
TAC	Transverse aortic constriction
LAD	Left anterior descending
SASP	Senescence-associated secretory phenotype
WGA	Wheat germ agglutinin
SD rats	Sprague–Dawley rats
LV	Left ventricle
γH2AX	Phosphorylated histone H2AX
